# Region-wise analysis of beef cow movements in Japan

**DOI:** 10.3389/fvets.2023.1012978

**Published:** 2023-02-02

**Authors:** Yoshinori Murato, Yoko Hayama, Sonoko Kondo, Kotaro Sawai, Emi Yamaguchi, Takehisa Yamamoto

**Affiliations:** Epidemiology Unit, National Institute of Animal Health, National Agriculture and Food Research Organization, Tsukuba, Japan

**Keywords:** animal infectious diseases, cattle movement, beef cow, Japan, tracing system

## Abstract

Animal movement is an important factor in the transmission of animal infectious diseases. A better understanding of movement patterns is therefore necessary for developing effective control measures against disease spread. In Japan, a cattle tracing system was established in 2003, following a bovine spongiform encephalopathy epidemic, and the information on all cattle movements has been stored in a national database maintained by the National Livestock Breeding Center. Using these data, we previously analyzed the movement of dairy cows, concluding that heterogeneities in cattle movement are associated with regional and seasonal factors. In the present study, we aimed to identify specific factors affecting the regional and seasonal movement patterns of beef cows in Japan. From April 2012 to March 2017, 797,553 farm-to-farm movement events were recorded. We analyzed movements by month and by cattle age and looked at the frequency of movement within and between seven regions spanning the national territory. Our results show that calf movement peaked at 9–10 months old; these movements were considered to be *via* the market and were frequent within and between regions. For inter-regional movements, Kyushu region was the top producer of calves for calf trading markets throughout Japan. With regard to intra-regional movements, round-trip movements for summer grazing were observed in May and October for cattle of various ages in the northern regions, especially Hokkaido and Tohoku. Moreover, the movements of Japanese Shorthorn breeds in Tohoku region exhibited consistent annual peaks in May and October/November, in accordance with their seasonal breeding practice. In the areas with high relative densities of dairy cows, such as Hokkaido, the shipping of newborn beef calves produced *via* embryo transfer to dairy cows was also observed. Overall, understanding the patterns of beef cow movement will help develop effective disease surveillance measures, such as pre-movement inspections focused on specific regions and types of movement.

## 1. Introduction

Animal movement represents a major means for the transmission of animal infectious diseases. Several important diseases, such as foot-and-mouth disease and tuberculosis, can spread through the movement of animals (1–4). A better understanding of animal movement is therefore essential for developing effective control measures against the spread of infections. Movement patterns are affected by factors such as species, breeds, and regions. Studies on animal movement have been conducted in some countries, including the United Kingdom and Australia, where regional and seasonal heterogeneities in the movement patterns of cattle were reported (5–8).

In Japan, all cattle numbers and their movements are to be reported into a cattle tracing system, which was established following the outbreak of bovine spongiform encephalopathy (BSE) in 2001. This system ensures that all cattle in Japan are registered with a unique number, and all movements from birth to death are recorded and stored in the database (9). In a previous study, we analyzed the movement of 1.36 million dairy cows, accounting for approximately one-third of the total cattle population in Japan, based on data from the cattle tracing system; we revealed heterogeneities in dairy cow movement, which were associated with regional and seasonal factors (10). However, the nationwide movement of beef cattle in Japan has not yet been studied.

In Japan, the primary breeds of beef and dairy cattle are Japanese Black and Holstein, respectively. The suitable environments for rearing these two breeds differ. Japanese Black cattle, which are relatively tolerant to high temperatures, are commonly raised in Kyusyu, which is located in the southern part of Japan, while Holsteins are commonly raised in Hokkaido, located in the northern part. Such differences in biological and geographical factors are expected to result in different movement patterns between beef and dairy cattle. In this study, we focused on breeding beef cattle, as they are expected to have more varied movement patterns because their rearing period is longer than that of feedlot cattle. Female beef cattle for breeding account for 35% of all beef cattle, including feeding cattle, whereas male breeding beef cattle account for <0.1%. Therefore, male breeding beef cattle were removed from this study because their movement patterns do not represent general trends (11, 12). Herein, we tried to determine specific factors which affect beef cow movement patterns regionally and seasonally.

## 2. Materials and methods

### 2.1. The national database of cattle information and movement record

Following the outbreak of BSE in September 2001, a cattle tracing system based on “the law for special measures concerning the management and relay of information for individual identification of cattle” was introduced throughout Japan in December 2003. As a result, all cattle are required to wear an ear tag with a unique individual identification number within 7 days of birth, and all movements from birth to death, including movements to slaughtering plants, are recorded and stored in a national database called the “Individual Cattle Identification Register (ICIR),” maintained by the National Livestock Breeding Center (NLBC). As required by law, all facilities involved in cattle movements, such as cattle farms, livestock markets, and slaughterhouses, must report all cattle movements to the NLBC with the following details: movement date, movement type (birth, transfer, and slaughter), and facility identification number. In this study we evaluated cattle movement data for 16 years from FY2005 (FY is the Japanese fiscal year, from April 1 to March 31) to FY2020 accumulated in the ICIR, along with cattle individual and facility information related to each movement. All data were obtained directly from the NLBC to the National Institute of Animal Health under the condition of “the Regulation for the Second Use of Individual Cattle Identification Register of National Livestock Breeding Center” and were anonymized by replacing farm- and cattle-identifiable data with randomized identification numbers before the analysis.

### 2.2. Preparation of data for analysis

Cattle movement data were connected to individual information and facility information *via* the individual identification number and facility number, respectively. Records of cattle with inaccurate movement histories, such as movement after death or before birth, were removed. Two movements connected by a stay of <1 day at any facility were converted to a single movement, considering that the movement was between farms, occurring *via* markets or traders. Individual information included individual identification number, date of birth, sex, breed, and the individual identification number of the mother. Cattle that appeared as mothers in the individual information for cattle involving whole movement data for 16 years were considered to have a history of calving at the birth date of the calf. Among female beef breed cattle, such as Japanese Black, those with at least one calving history as of March 31, 2021 (the last date of the study period) were considered beef cows. However, lifetime calving history was used as a criterion to identify a mother cow, because the feeders were excluded in this study; cattle younger than the expected age of their first delivery at the end of the study period could not be correctly classified. Since the 95th percentile of the age at first delivery was 36 months, movement records in the last 3 years (FY2018 to FY2020) were excluded. Additionally, in Japan, several major events influencing cattle movement have occurred in recent years, including the outbreak of foot-and-mouth disease in 2010 and the Great East Japan Earthquake in 2011. As this study aimed to reveal general characteristics of cattle movement, we focused on the period after FY2012, that is, a period without any major accidental events influencing livestock movements. Consequently, beef cow movement records within the 6 years from FY2012 to FY2017 were included in the analysis.

### 2.3. Regional-level movement

**Figure 1:**
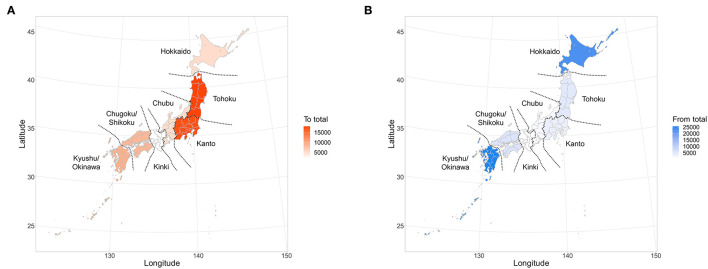
Classification of Japanese regions in this study and total number of movements of beef cows toward each region **(A)** and from each region **(B)**.

In this study, “between-farm movement” records were extracted by removing “births (including imports)” and “deaths (including slaughter)” from the dataset. For between-farm movement, departure and arrival farms were classified into the following seven regions: Hokkaido (HKD), Tohoku (THK), Kanto (KTO), Chubu (CHU), Kinki (KNK), Chugoku/Shikoku (C_S), and Kyushu/Okinawa (K_O), as shown in Figure 1, according to their locations. The number of between-farm movements of beef cows was tabulated by the departure and arrival regions.

### 2.4. Month at the time of movement

The number of monthly inter-regional and intra-regional movements of beef cows was counted. When the number of monthly movements suggested possible seasonality, time series analysis was conducted by plotting seasonal subseries. In the seasonal subseries plotting, the length of the seasonal pattern was defined as a year and the subseries data for each month are plotted side by side horizontally. Regarding intra-regional movements, the number of movements per month was also counted by region.

### 2.5. Age in months at the time of movement

The number of movements by age in months was counted for inter-regional and intra-regional beef cow movements, respectively. When the number of movements by age was particularly skewed toward specific ages, further analysis, such as tabulating by the departure and arrival regions, was conducted focusing on the ages of interest. Additionally, the number of movements by age of month was counted by the calendar month. Regarding the intra-regional movements, the number of movements by age in months was also counted by region. When the number of movements by age showed a unique pattern for a specific region, further analysis, such as counting by the calendar month and breed, was conducted focusing on the regions of interest. All analyses were conducted using R version 4.0.5. with the forecast package for time series analysis.

## 3. Results

### 3.1. Overview of the beef cow population in Japan

A total of 700,000 beef cows of all ages were raised in Japan, of which 380,000 (55%) were kept in Kyushu/Okinawa (Supplementary Figure 1). The demographics of beef cows born in FY2017 are described in Table 1, in terms of region and breed of the mother cow. A total of 72,000 beef cows were born in Japan, of which 41,000 (57%) were born in Kyushu/Okinawa.

**Table 1:** The number of beef cow births by region and breed of mother cow in Japan in FY2017.

**Region**	**Number of cattle (Proportion for each region)**						**Total number of cattle (Proportion for grand total)**	
	**Breed type of mother cow**							
	**Beef breed**		**Dairy breed**		**Crossbreed**			
Hokkaido	7,902	(80.8%)	1,260	(12.9%)	622	(6.4%)	9,784	(13.6%)
Tohoku	8,876	(93.0%)	529	(5.5%)	137	(1.4%)	9,542	(13.2%)
Kanto	3,141	(76.1%)	885	(21.4%)	100	(2.4%)	4,126	(5.7%)
Chubu	1,158	(74.6%)	344	(22.2%)	51	(3.3%)	1,553	(2.2%)
Kinki	1,695	(93.9%)	39	(2.2%)	71	(3.9%)	1,805	(2.5%)
Chugoku/Shikoku	3,502	(87.7%)	419	(10.5%)	74	(1.9%)	3,995	(5.5%)
Kyushu/Okinawa	39,910	(96.5%)	957	(2.3%)	470	(1.1%)	41,337	(57.3%)
Total	66,184	(91.7%)	4,433	(6.1%)	1,525	(2.1%)	72,142	(100%)

Crossbred, or F1 cattle, are calves derived from dairy cows with beef breed semen.

### 3.2. Overview of between-farm movement

**Figure 2:**
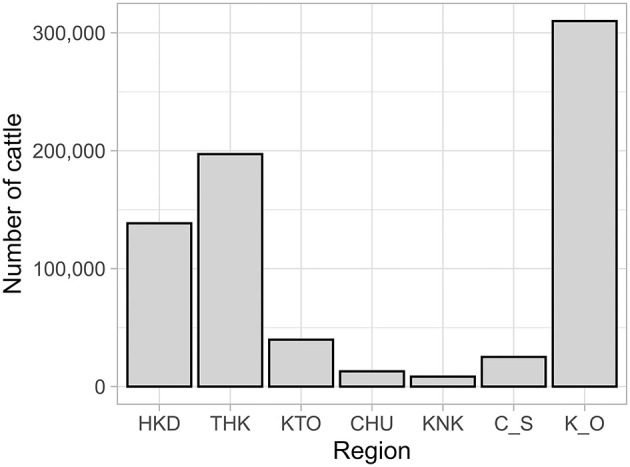
Number of intra-regional movements of beef cows by region in Japan from FY2012 to FY2017. HKD, Hokkaido; THK, Tohoku; KTO, Kanto; CHU, Chubu; KNK, Kinki; C_S, Chugoku/Shikoku; K_O, Kyushu/Okinawa.

A total of 797,553 between-farm movements were recorded for beef cows in the period from FY2012 to FY2017. The number of movements per year remained relatively constant across the study period, ranging from 124,610 to 138,676, with an average of 133,000. The numbers of regional-level movements are shown in Table 2 and Figure 2 for inter- and intra-regional movements, respectively. Ninety-two percent (731,871) of all farm-to-farm movements were intra-regional, and 8% (65,682) were inter-regional.

**Table 2:** The number of inter-regional movements of beef cows.

**Departure region (from)**	**Arrival region (to)**							**From total**
	**Hokkaido**	**Tohoku**	**Kanto**	**Chubu**	**Kinki**	**Chugoku/Shikoku**	**Kyushu/Okinawa**	
Hokkaido	- -	8,356 (12.7%)	9,848 (15.0%)	1,545 (2.4%)	124 (0.2%)	522 (0.8%)	2,689 (4.1%)	**23,084** **(35.1%)**
Tohoku	688 (1.0%)	- -	2,277 (3.5%)	441 (0.7%)	83 (0.1%)	250 (0.4%)	1,193 (1.8%)	**4,932** **(7.5%)**
Kanto	849 (1.3%)	1,419 (2.2%)	- -	978 (1.5%)	111 (0.2%)	105 (0.2%)	274 (0.4%)	**3,736** **(5.7%)**
Chubu	294 (0.4%)	281 (0.4%)	680 (1.0%)	- -	187 (0.3%)	157 (0.2%)	324 (0.5%)	**1,923** **(2.9%)**
Kinki	19 (0.0%)	70 (0.1%)	35 (0.1%)	85 (0.1%)	- -	172 (0.3%)	192 (0.3%)	**573** **(0.9%)**
Chugoku/Shikoku	270 (0.4%)	236 (0.4%)	254 (0.4%)	172 (0.3%)	254 (0.4%)	- -	4,703 (7.2%)	**5,889** **(9.0%)**
Kyushu/Okinawa	3,127 (4.8%)	7,642 (11.6%)	4,114 (6.3%)	2,025 (3.1%)	1,159 (1.8%)	7,478 (11.4%)	- -	**25,545** **(38.9%)**
**To total**	**5,247** **(8.0%)**	**18,004** **(27.4%)**	**17,208** **(26.2%)**	**5,246** **(8.0%)**	**1,918** **(2.9%)**	**8,684** **(13.2%)**	**9,375** **(14.3%)**	**65,682** **(100.0%)**

The number of movements by beef cows between each departure and arrival region in Japan from FY2012 to FY2017 are shown. The bold values indicate the number (or %) for the total.

### 3.3. Inter-regional movement

**Figure 3:**
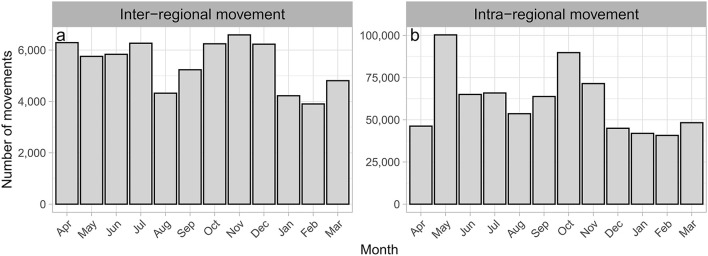
Number of monthly movements of beef cows. Inter-regional **(a)** and intra-regional movements **(b)** of beef cows in Japan from FY2012 to FY2017.

**Figure 4:**
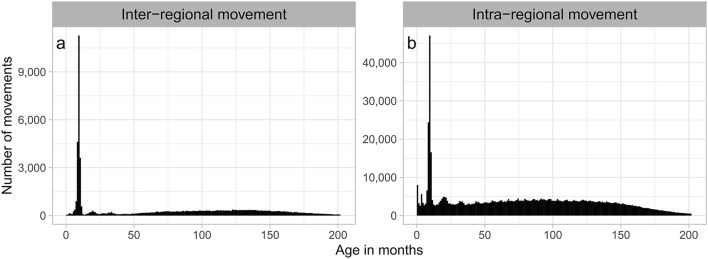
Age distribution of beef cows that moved within regions **(a)** and between regions **(b)** in Japan from FY2012 to FY2017.

The number of inter-regional movements per month was lower in August-September (7–8%) and January-March (6–7%) than in other months (9–10%; Figure 3a). This seasonality was observed throughout the study period (Supplementary Figure 2A). The age distribution of inter-regional movements peaked at 9–10 months of age, and this peak was observed for all seasons (Supplementary Figure 3). Movements at 8–11 months of age accounted for 30% of all inter-regional movements (Figure 4a), with 69% of these movements originating in Kyusyu/Okinawa (Table 3).

**Table 3:** The number of inter-regional movements of beef cows from 8 to 11 months old.

**Departure region (from)**	**Arrival region (to)**							**From total**
	**Hokkaido**	**Tohoku**	**Kanto**	**Chubu**	**Kinki**	**Chugoku/Shikoku**	**Kyushu/Okinawa**	
Hokkaido	- -	1,228 (6.3%)	236 (1.2%)	221 (1.1%)	13 (0.1%)	148 (0.8%)	203 (1.0%)	**2,049** **(10.5%)**
Tohoku	129 (0.7%)	- -	371 (1.9%)	207 (1.1%)	50 (0.3%)	104 (0.5%)	160 (0.8%)	**1,021** **(5.2%)**
Kanto	119 (0.6%)	856 (4.4%)	- -	218 (1.1%)	7 (0.0%)	19 (0.1%)	170 (0.9%)	**1,389** **(7.1%)**
Chubu	74 (0.4%)	135 (0.7%)	77 (0.4%)	- -	63 (0.3%)	49 (0.3%)	106 (0.5%)	**504** **(2.6%)**
Kinki	1 (0.0%)	45 (0.2%)	8 (0.0%)	10 (0.1%)	- -	15 (0.1%)	101 (0.5%)	**180** **(0.9%)**
Chugoku/Shikoku	175 (0.9%)	131 (0.7%)	132 (0.7%)	111 (0.6%)	74 (0.4%)	- -	333 (1.7%)	**956** **(4.9%)**
Kyushu/Okinawa	2,497 (12.8%)	6,179 (31.6%)	1,407 (7.2%)	1,301 (6.7%)	875 (4.5%)	1,172 (6.0%)	- -	**13,431** **(68.8%)**
**To total**	**2,995** **(15.3%)**	**8,574** **(43.9%)**	**2,231** **(11.4%)**	**2,068** **(10.6%)**	**1,082** **(5.5%)**	**1,507** **(7.7%)**	**1,073** **(5.5%)**	**19,530** **(100.0%)**

The number of movements by beef cows between each departure and arrival region in Japan from FY2012 to FY2017 are shown. The bold values indicate the number (or %) for the total.

### 3.4. Intra-regional movement

**Figure 5:**
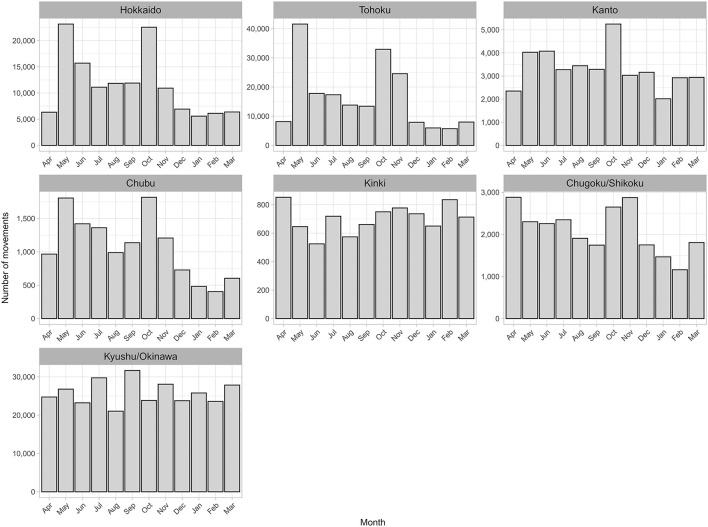
Number of intra-regional movements of beef cows by region and by month in Japan from FY2012 to FY2017 by region.

**Figure 6:**
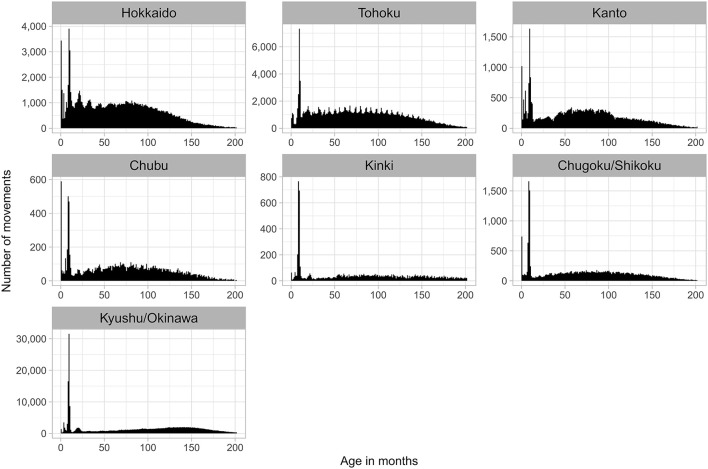
Age distribution of beef cows that moved within regions in Japan from FY2012 to FY2017 by region.

The number of monthly intra-regional movements was higher in May (14%) and October (12%) than in other months (6–10%; Figure 3a), and this seasonality was observed throughout the study period (Supplementary Figure 2B). Comparing the number of movements per month by region, peaks were clearly observed in May and October in all regions other than Kinki and Kyushu/Okinawa, especially in Hokkaido and Tohoku, which are located in the northern part of Japan (Figure 5). To examine the characteristics of movements in May and October, we analyzed these at the individual level using FY2017 data. Consequently, 38% of cows in Hokkaido that moved in May also moved in October, accounting for 34% of those that moved in October. In Tohoku, 39% of the cows that moved in May also moved in October, accounting for 49% of those moving in October. Comparing the age distribution at intra-regional movement by region, a peak at 8–10 months was observed in all regions (Figure 4b, Supplementary Figure 3), and another peak at <1 month was observed in Hokkaido, Kanto, Chubu, and Chugoku/Shikoku (Figure 6). Additionally, the age distribution for movement within Tohoku showed several small peaks, with intervals of 5–7 months over almost 150 months of age (Figure 6). When the distribution of age at movement within Tohoku was broken down by month, peaks with 12-month intervals were observed in May, October, and November, and the difference between peaks of the age at the movement in May and October/November was about 5 months (Supplementary Figure 4). To examine the reason for these peaks with 12-month intervals, we compared the age distribution at movement by breed. The peaks with 12-month intervals were only observed for Japanese Shorthorn cattle (Supplementary Figure 5).

## 4. Discussion

The analysis of beef cow movements revealed that the frequency of movement varied depending on the season, age, rearing area, and breed. Further, these characteristics differed from the ones observed for dairy cows.

Regarding the distribution of age at the time of movement, a peak of high frequency of movements was observed for calves around 9–10 months of age, visible in every month of the year, irrespective of movement type (inter- or intra-regional). The beef-calf markets in Japan generally deal with calves aged about 8–10 months, be it for breeding or fattening (13, 14). This suggests that these movements mainly represent the movement of calves through markets. With regard to inter-regional movements, two-thirds or more of these were of calves departing from Kyushu/Okinawa, which accounted for more than half of both the number of beef cows raised in Japan and newborn beef cows, highlighting Kyushu/Okinawa as the major supplier of replacement beef cows to other regions. Meanwhile, Hokkaido, which holds the largest share of dairy cows kept and born in Japan, exported the largest number of replacement dairy cows to other regions (93%), highlighting its significance as a source of replacement dairy cows (10).

Peaks of high frequency of movements for calves at <1 month of age were observed in the age distribution for intra-regional movements in Hokkaido, Kanto, Chubu, and Chugoku/Shikoku. The proportion of beef calves delivered from dairy cows among all beef calves was relatively high in these regions. In Japanese dairy farms, it is common practice to transfer beef cattle embryos, usually Japanese Black embryos, into dairy cows in order to produce beef calves while inducing the lactation of delivered dairy cows (13, 15). Although a similar practice can be performed *via* the artificial insemination of dairy cows with beef breed semen, the produced beef cow calves are crossbred (also called F1 cattle), and this study is targeting the movements of beef breeding cows. Thus, these are considered movements of newborn beef calves produced *via* embryo transfer to dairy cows.

As for the number of monthly movements, lower inter- and intra-regional movement numbers were commonly observed in the summer and winter months. Shipping animals potentially causes physical and mental stress, which could in turn reduce productivity. This may explain the seasonality of the movement, as movement is avoided during the hot summer months and cold winter months to minimize such shipping stress. The number of intra-regional movements was much higher in May and October than in any other month. Moreover, in Hokkaido and Tohoku, where such seasonality was clearly observed, over one-third of intra-regional movements in May and October were conducted by the same cattle. Thus, these movements were likely round-trip movements. In Japanese cattle farming, summer grazing is commonly practiced, as cattle are transferred to the pasture in May, when the grassland becomes available, and leave the pasture around October, before the snowfall (16, 17). This indicates that the seasonality observed is due to summer grazing and is represented by movements between the source farms and common pastures. However, in Kyushu/Okinawa, which is located in the southern part of Japan with a relatively high average temperature, this seasonality was not clearly observed, presumably because year-round grazing is possible (17). In Kinki, which hosts only 0.1% of the total pastureland area in Japan, the smallest share compared to other regions (0.6–84%) (18), it is possible that summer grazing is not actively practiced, and this may be a reason for the lack of a clear seasonality. Although the total number of monthly intra-regional movements was higher in May and October, the proportion of movements between 8 and 11 months of age was lower in these 2 months than in the other months. This indicates that the higher number of intra-regional movements in May and October was not mainly due to shipping calves to the market. In addition, the age distribution of beef cow that moved in both May and October 2017 indicated that these movements were not limited to heifers up to the average age of the first calving in beef cows (24.5 months old) in Japan (19), but was observed over a wide range of ages. Meanwhile, similar seasonality in cattle movements due to summer grazing was also observed in Japanese dairy cows (10). During the summer grazing of dairy cows, peaks in the age distribution at movement in May and October were observed at 13–14 and 19–20 months of age and thus were considered as the movement of growing heifers. This difference in the age of summer grazing movement between beef and dairy cows may result from the fact that the latter are rarely grazed after first calving because of milking, whereas beef cows do not need a milking period and can perform summer grazing regardless of age or calving number.

In the age distribution at movement within Tohoku, additional small peaks in small increments repeatedly appeared only in May, October, and November, and these peaks were 12 months of age apart, suggesting that cows forming these peaks may partake in summer grazing. Moreover, we found that Japanese Shorthorns formed these peaks. The Japanese Shorthorn is a breed of Wagyu cattle that has been improved to be suitable for summer grazing by crossing the Nanbu cattle, a native breed raised in the highlands in Tohoku, with the imported Shorthorn breed (20–23). Japanese Shorthorn calves and their dam are usually extensively raised in Tohoku, with dam-calf summer grazing traditionally implemented to save labor. To allow mother-calf pairs to spend their suckling period in the summer pasture, Japanese Shorthorns have been subjected to seasonal breeding so that calves are born around March, prior to May when they are transferred to the pasture. Therefore, the clear peaks with annual intervals in Japanese Shorthorns can be explained by this seasonal breeding management followed by summer grazing every year throughout their lives.

In addition, as it provides insight into the movement heterogeneity of Japanese beef cattle, this study will also be useful for planning the surveillance of specific animal diseases. In Japan, chronic infectious diseases of cattle causing low productivity, such as enzootic bovine leukosis (EBL) and bovine viral diarrhea (BVD), endemically occur each year (24). Transmission of EBL and BVD is mainly caused by cohabitation with infected cattle, and thus the introduction of cattle from other farms has been reported as a risk factor for disease introduction (25, 26). For example, summer grazing has been reported to be an important risk factor for the transmission of BVD (26, 27). As this study revealed that summer grazing is more frequent in northern regions such as Hokkaido and Tohoku, inspections of cattle moving for summer grazing are encouraged in these regions. Similarly, regarding the inter-regional movements, the movement of 9–10-month-old calves was demonstrated to most frequently occur from Kyushu/Okinawa in this study. This suggested that the surveillance of calves shipped to the market in this region may effectively contribute to the suppression of trans-regional between-farm transmission. In addition, the expected number of samples and the human and budgetary resources required for these surveillances could be also driven by our results.

The results of this study revealed age-dependent movement, i.e., shipments of calves *via* markets, and season-dependent round-trip movement of the same cow, i.e., entering and returning into/from pasturelands during summer grazing. With regard to inter-regional calf shipment, Kyushu/Okinawa emerged as the most important supplier. Meanwhile, summer grazing was more commonly practiced in the northern regions, especially in Hokkaido and Tohoku. We also observed unique movement of Japanese Shorthorns in the Tohoku region and characteristic movement of newborn beef calves produced *via* embryo transfer to dairy cows. The findings regarding these heterogeneities in cattle movements depending on the season, age, or cattle breed, as identified in this study, will help develop effective disease surveillance measures, such as pre-movement inspections focused on specific regions and movement types.

## Data availability statement

The data that support the findings of this study are available from the National Livestock Breeding Center (https://www.nlbc.go.jp/); however, restrictions apply to the availability of these data, which were used under license for the current study, and are, therefore, not publicly available.

## Author contributions

TY conceived and designed the study. YM and TY collected the data. YM, YH, and TY analyzed the data. SK, KS, and EY contributed to the interpretation of the results. YM drafted the manuscript. KS, YH, and TY revised the main manuscript text. All authors have read and approved the final manuscript.
