# Supplementary material for: Region-wise analysis of beef cow movements in Japan
Source: Front Vet Sci. 2023 Feb 2;10:1012978. doi: 10.3389/fvets.2023.1012978 (PMC9932799; doi:10.3389/fvets.2023.1012978)
Supplement: Supplementary file 1 [file Data_Sheet_1.docx]

Supplementary Material

# Supplementary Figures


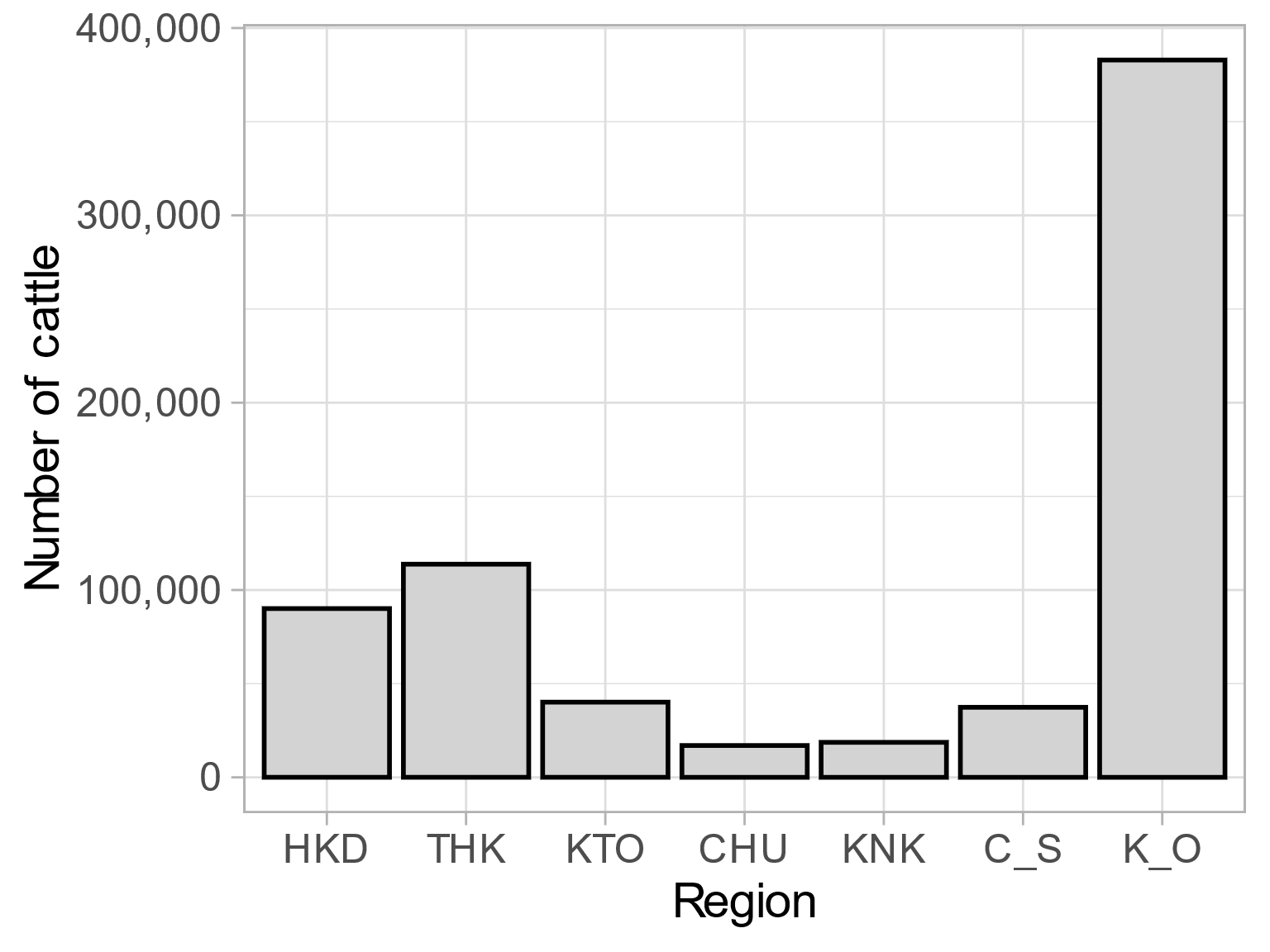


**Supplementary Figure 1.** Number of beef cows by region in Japan as of April 1, 2017. (HKD: Hokkaido, THK: Tohoku, KTO: Kanto, CHU: Chubu, KNK: Kinki, C_S: Chugoku/Shikoku, K_O: Kyushu/Okinawa)


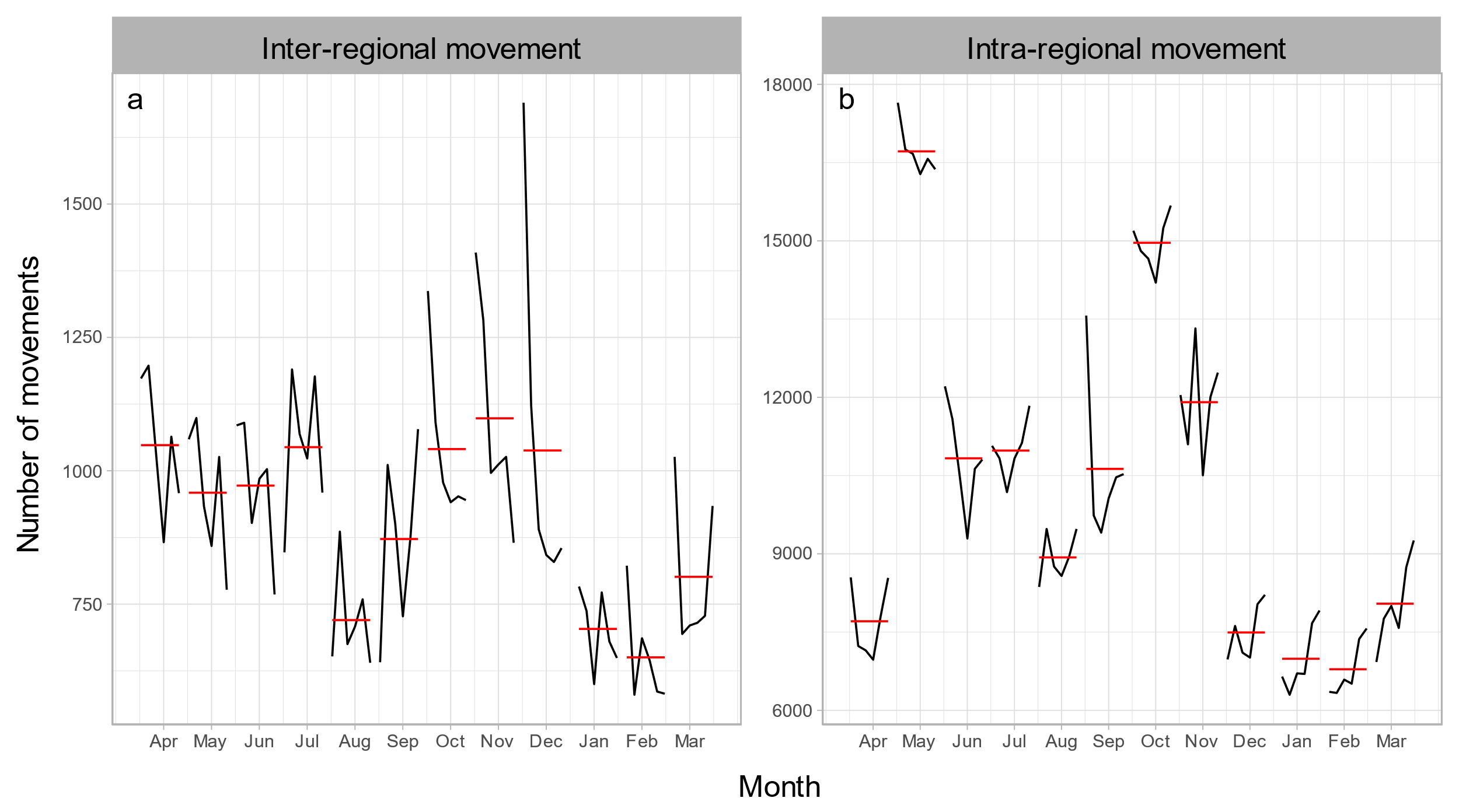


**Supplementary Figure 2.** Time series analysis of monthly movements of beef cows. Seasonal trend by months throughout the year is extracted by plotting monthly subseries plot. Inter-regional (a) and intra-regional movements (b) of beef cows in Japan from FY2012 to FY2017.


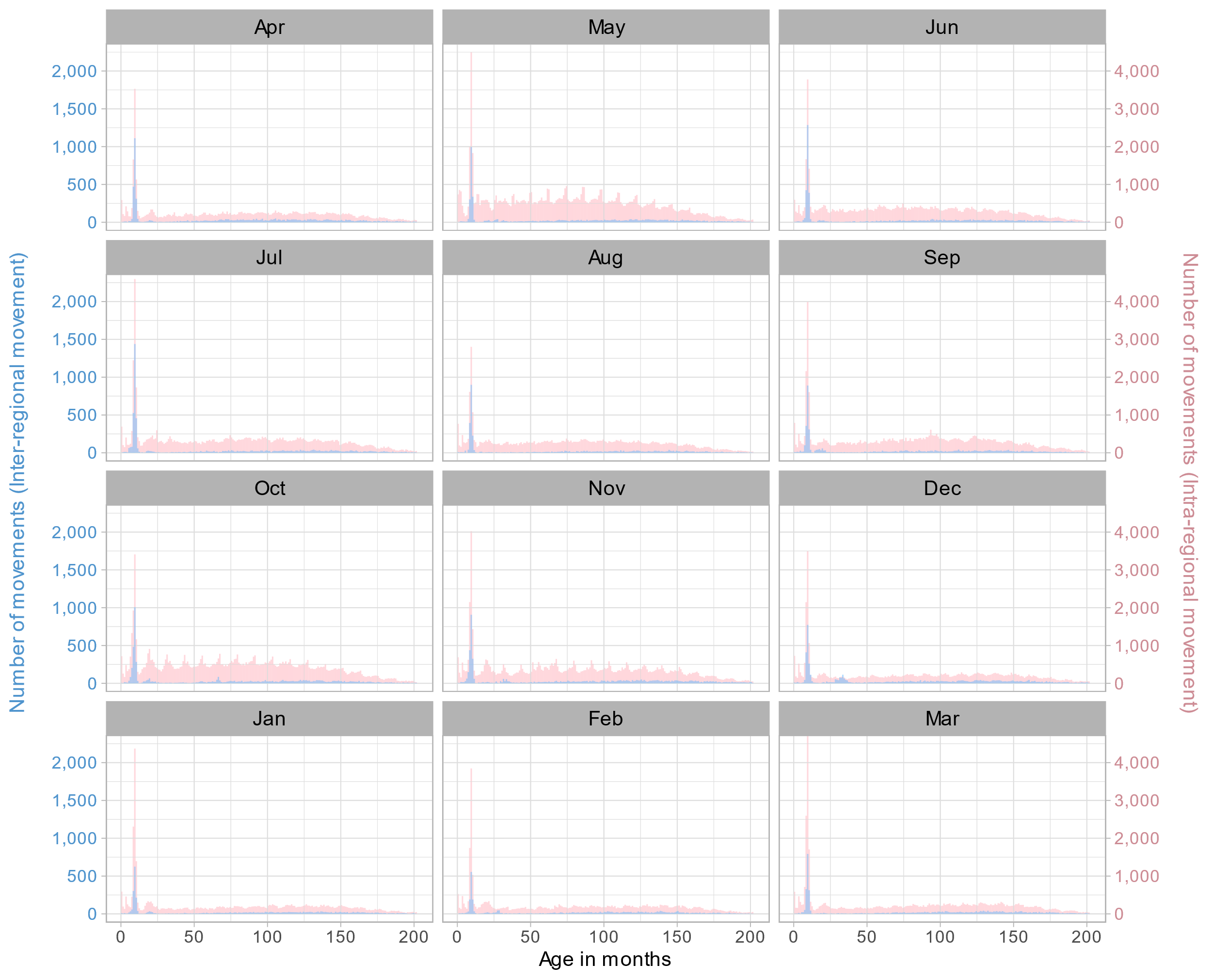


**Supplementary Figure 3.** Age distribution of beef cows in Japan from FY2012 to FY2017 by month. Inter-regional movements are shown in blue and intra-regional movements are shown in pink.


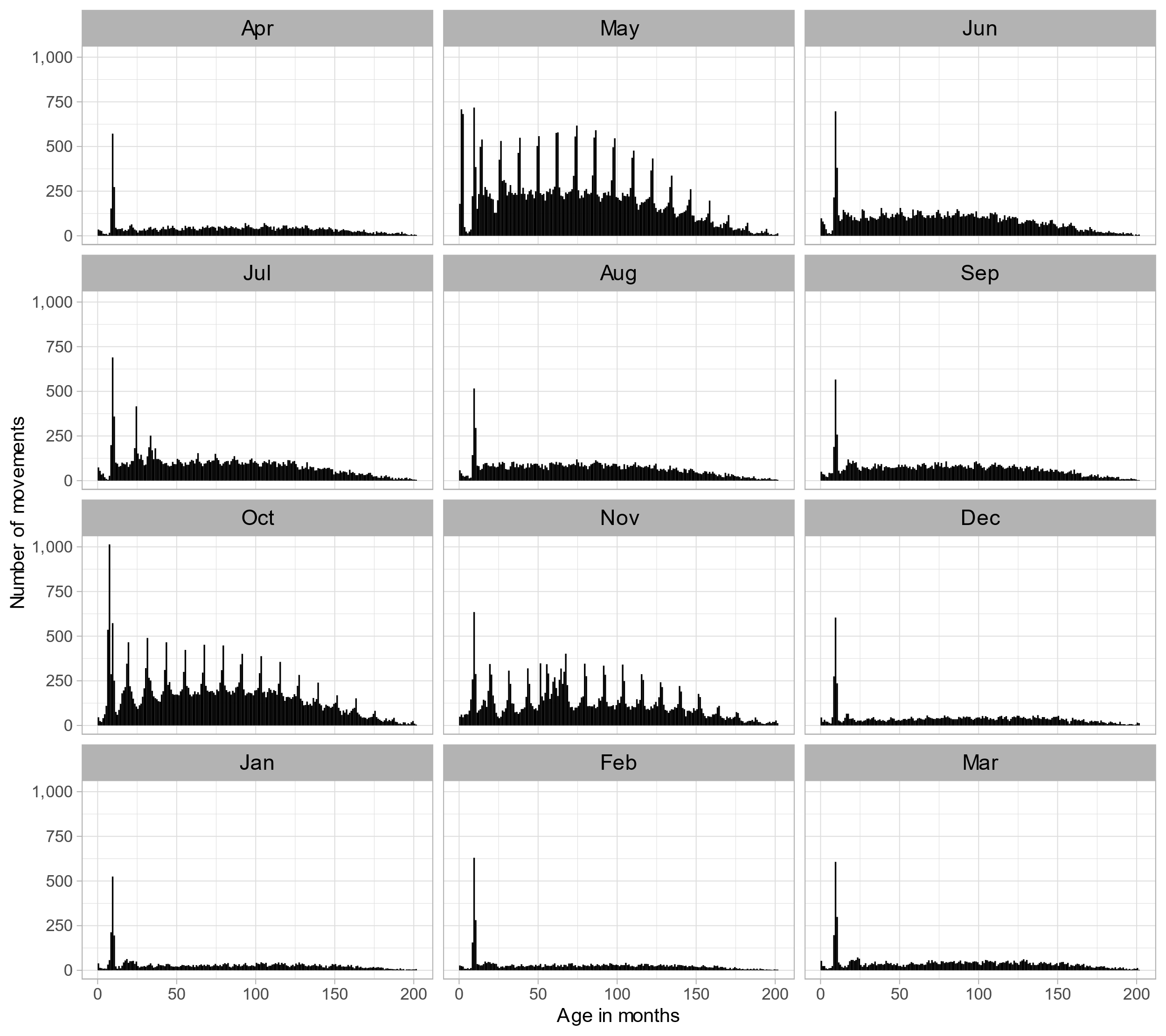


**Supplementary Figure 4.** Age distribution of beef cows moved within Tohoku from FY2012 to FY2017 by month.


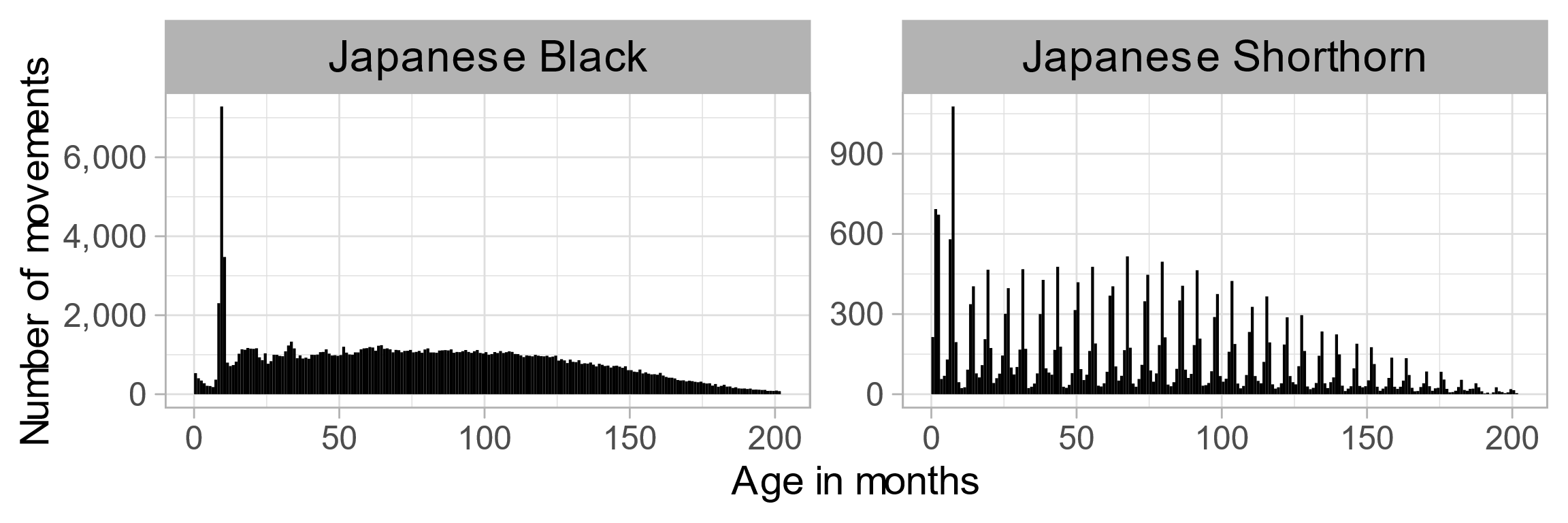


**Supplementary Figure 5.** Age distribution of beef cows moved within Tohoku from FY2012 to FY2017 by breed. Breeds accounting for less than 1% of the total beef cow population in Tohoku as of April 1, 2017, were not plotted.
